
JOURNAL INFORMATION
==============================
NLM Title Abbreviation: Tex Med J (Austin)
NLM Journal ID: 21130590R

Texas Medical Journal

ISSN: 0892-8495
Publisher: Biomedical Journal Digitization Project

ARTICLE INFORMATION
==============================
PMCID: PMC9649183
PMID: 36957541
Article version: 1
Subjects: Abstracts and Selections

Powerful Sick

Print publication date: 1917 Feb
Volume: 32
Issue: 8
First page: 377
Last page: 377
License: This work is free of known copyright restrictions. For more information, please see PMC Back Issue Digitization.
License URL: https://creativecommons.org/publicdomain/mark/1.0/

==============================

wee bit "glib o' the gab." The doctor then, feeling he had him, bluntly asked:

"How does this square wi' your teetotal pretentions?"

"Weel," quoth John, with a quiet smile, "though I'm a stanch teetotaler, I'd be a fule to refuse what the doctor orders."—*Pittsburg Chronicle Telegraph.*

---

### "Powerful Sick."

In the bright sunlight on a railroad station in Georgia slept a colored brother. He snored gently, with his mouth ajar and his long, moist tongue resting on his chest like a pink plush necktie. A Northerner climbed off a train to stretch his legs, unscrewed the top of a capsule and, advancing on tip-toe, dusted the grains of quinine on the surface of the darkey's tongue. Presently the negro sucked his tongue back inside his mouth and instantly he arose with a start and looked about him wildly.

"Mistah," he said to the joker, "is you a doctah?"

"Nope."

"Well, then, kin yo' tell me whar I kin fin' me a doctah right away?"

"What do you want with a doctor?"

"I'm sick."

"How sick?"

"Powerful sick."

"Do you know what's the matter with you?"

"Suttin'ly I knows wut's de matteh with me—mah gall's busted!"—*Selected.*

---

### Helpful Hints for the Busy Doctor.

THE SEQUELAE OF LA GRIPPE.—Among all of the various acute and exhausting illnesses that afflict mankind, there is none that so generally results in distinct prostration as epidemic influenza, or la grippe. Even the grippal infections which are uncomplicated or unaccompanied by serious organic changes are more than apt to leave the patient in a thoroughly devitalized condition after the acute febrile symptoms have subsided. It is for this reason that the treatment of la grippe convalescence is of special importance. The anemic, debilitated, depressed patient requires a systemic "booster" that will not only stimulate but revivify and reconstruct. It is distinctly wise, in such cases, to commence vigorous tonic treatment as early as possible, preferably by means of Pepto-Mangan (Gude), the hemic builder and general reconstituent. This standard hematinic increases the vital elements of the circulating blood and, by increasing the appetite and improving the absorptive and assimilative functions, quickly restores both hemic and general vitality.

---

ARE YOU SEEKING A RELIABLE TONIC?—Conservative medical men are neither asked nor expected to accept the opinions or conclusions of anyone else concerning the value of Gray's Glycerine Tonic Comp. The only request of the manufacturers is that the physician who is seeking a tonic, a dependable means of restoring the activity of the bodily functions, will give this remedy a fair and reasonable trial. To his conclusions as to the results obtained—his judgment as to the superiority of this remedy as a means of overcoming debility, inanition and malnutrition—the de-
